# Retraction Note to: Methylene blue photochemical treatment as a reliable SARS-CoV-2 plasma virus inactivation method for blood safety and convalescent plasma therapy for COVID-19

**DOI:** 10.1186/s12879-021-06344-9

**Published:** 2021-07-09

**Authors:** Changzhong Jin, Bin Yu, Jie Zhang, Hao Wu, Xipeng Zhou, Hangping Yao, Fumin Liu, Xiangyun Lu, Linfang Cheng, Miao Jiang, Nanping Wu

**Affiliations:** 1 State Key Laboratory for Diagnosis and Treatment of Infectious Diseases, National Clinical Research Center for Infectious Disease, National Medical Center for Infectious Disease, Collaborative Innovation Center for Diagnosis and Treatment of Infectious Diseases, The First Affiliated Hospital, School of Medicine, Zhejiang University, Hangzhou, 310003 China; 2 Boxin (Beijing) Biotechnology Development LTD, 4/F, Tower B, Siemens Building, No. 7 South Central Road, Wangjing, Chaoyang District, Beijing, China; 3 Ministry of Education Key Lab of Hazard Assessment and Control in Special Operational Environment, Preventive Medicine Institute, Air force Medical University, Xi’an, China

**Retraction Note: BMC Infect Dis 21, 357 (2021)**

**https://doi.org/10.1186/s12879-021-05993-0**

The editor has retracted this article. Following publication concerns were raised regarding Fig. 1, specifically:

- The Light 0 min panel for Virus Control appears to partially overlap with the Light 0 min panel for 1uM MB No light.
- The Light 0 min panel for 1um MB No light appears to partially overlap with the Light 40 Min panel of the same row, and partially overlap with the Light 0 Min panel for No MB.

In addition, the authors Bin Yu, Jie Zhang, Hao Wu, Xipeng Zhou and Miao Jiang did not appropriately declare a competing interest regarding their affiliation with Boxin (Beijing) Biotechnology Development LTD and its product ‘BX-1 AIDS treatment instrument’ as described in this article.

The editor therefore no longer has confidence in the reliability of the data reported in the article.

Changzhong Jin, Bin Yu, Jie Zhang, Hangping Yao, Fumin Liu, Xiangyun Lu, Linfang Cheng, Miao Jiang and Nanping Wu do not agree to this retraction. Hao Wu and Xipeng Zhou have not responded to any correspondence from the editor about this retraction.

